# Irony of Iron Overload: Hereditary Hemochromatosis Complicated by Alcoholic Hepatitis, Gastrointestinal Bleeding, and Intramuscular Hematoma

**DOI:** 10.7759/cureus.105412

**Published:** 2026-03-17

**Authors:** Hans Jesper F Del Mundo, Adelaine Joy Espiritu, Beatrice Elizabeth Ong, Harold Manansala, Hardikkumar Bhanderi

**Affiliations:** 1 Internal Medicine, Monmouth Medical Center, Long Branch, USA

**Keywords:** dieulafoy lesion, gastrointestinal bleeding (gib), h63d variant, hereditary hemochromatosis (hh), intramuscular hematoma, severe alcoholic hepatitis

## Abstract

Hereditary hemochromatosis (HH) is an autosomal recessive disorder characterized by excessive iron absorption and accumulation. The *HFE* gene C282Y mutation is most commonly implicated, while the H63D variant is less penetrant and rarely causes significant iron overload. However, alcohol use accelerates hepatic iron accumulation and worsens liver injury, creating a complex interplay between genetic and environmental factors. A 47-year-old woman with chronic heavy alcohol use presented with progressive jaundice and generalized weakness. Laboratory evaluation revealed markedly elevated aspartate aminotransferase (AST) of 542 U/L, alanine aminotransferase (ALT) of 185 U/L, total bilirubin of 22.2 mg/dL, and ferritin of 17,741 ng/mL. Imaging demonstrated hepatomegaly with coarse echotexture suggestive of cirrhosis. Genetic testing identified heterozygosity for the H63D variant of the *HFE* gene. She was initially treated for severe alcoholic hepatitis with oral prednisolone based on an elevated Maddrey’s Discriminant Function (MDF) score of 73.4; however, therapy was discontinued after the Lille score exceeded 0.45 at day 7, indicating poor response to corticosteroids. Her hospital course was complicated by recurrent gastrointestinal (GI) bleeding due to esophageal ulceration, esophageal varices, portal hypertensive gastropathy, and a Dieulafoy lesion requiring endoscopic clipping and band ligation. She also developed a large intramuscular hematoma in the left vastus lateralis muscle, requiring interventional drainage and multiple blood transfusions. She was ultimately discharged to a subacute rehabilitation facility for alcohol detoxification with plans for close outpatient follow-up with gastroenterology and hepatology. This case highlights the paradoxical coexistence of extreme hyperferritinemia with severe anemia in advanced liver disease complicated by recurrent hemorrhage. The markedly elevated ferritin likely reflected a combination of hepatic inflammation, alcohol-related injury, and dysregulated iron metabolism rather than primary genetic hemochromatosis. Although the H63D variant alone rarely leads to significant clinical disease, chronic alcohol use may contribute to worsening hepatic dysfunction and iron imbalance. Additionally, the presence of multiple GI bleeding sources and a spontaneous intramuscular hematoma illustrates the complex hemostatic disturbances associated with advanced cirrhosis. Early recognition of atypical bleeding manifestations in patients with decompensated liver disease is essential to guide timely intervention and improve clinical outcomes.

## Introduction

Hereditary hemochromatosis (HH) is a common genetic disorder of iron metabolism that affects approximately 1 in 200-400 individuals. It is inherited in an autosomal recessive pattern, most often due to mutations in the HFE gene. These mutations alter the regulation of the transferrin receptor and downregulate hepcidin expression, leading to increased intestinal iron absorption and excessive iron recycling from senescent red blood cells [1]. The liver, which plays a central role in iron regulation, is particularly vulnerable to injury from iron overload. When serum ferritin levels exceed 1000 µg/L, the risk of cirrhosis is significantly increased. The most common pathogenic variant is homozygosity for the C282Y mutation, while the H63D variant is less frequently associated with clinically significant iron overload. Individuals homozygous for H63D or heterozygous carriers generally have a lower risk of progressive disease compared with C282Y homozygotes or compound heterozygotes (C282Y/H63D) [2]. Clinical manifestations of HH are often nonspecific, including fatigue, hepatomegaly, and abnormal liver function tests, but advanced iron overload may involve cardiac, hepatic, and endocrine complications.

Alcoholic hepatitis (AH), in contrast, is an acute inflammatory condition of the liver resulting from chronic and heavy alcohol use. It typically presents with jaundice, malaise, and tender hepatomegaly, accompanied by moderately elevated aminotransferases with an aspartate aminotransferase (AST)-to-alanine aminotransferase (ALT) ratio ≥2, hyperbilirubinemia, and coagulopathy [3]. The threshold of alcohol consumption required to trigger AH remains unclear, but most patients have a history of sustained, excessive intake, often over decades [4]. Prognostic scoring systems such as Maddrey’s Discriminant Function (MDF) and the Model for End-Stage Liver Disease (MELD) help guide disease severity assessment and treatment decisions. Corticosteroids are recommended for severe cases, and treatment response is monitored using the Lille score after seven days [3].

## Case presentation

A 47-year-old female with a past medical history of alcohol use disorder and depression presented with a five-day history of jaundice and generalized weakness. Laboratory evaluation revealed serum alcohol 53.5 mg/dL and serum ammonia 62 µmol/L. Significant derangements in liver function test results were noted on admission, including AST 542 U/L, ALT 185 U/L, total bilirubin 22.2 mg/dL (direct 15.2 mg/dL), and alkaline phosphatase 428 U/L, as well as electrolyte abnormalities, including hyponatremia and hypokalemia. A complete blood count showed normocytic anemia with hemoglobin 11.4 g/dL. Furthermore, an MDF score was found to be elevated at 73.4. These findings were initially attributed to her significant alcohol use history, and she was treated for suspected severe alcoholic hepatitis, which was later complicated by hepatic encephalopathy. She was managed with intravenous N-acetylcysteine for five doses, oral prednisolone for seven days, rifaximin, lactulose, and was placed on a CIWA protocol. After seven days of steroid administration, the Lille score was found to be > 0.45, and prednisolone was discontinued. A routine anemia workup was also pursued, which revealed a significantly elevated ferritin level of 17,741 ng/mL, prompting consideration of other etiologies, including hereditary hemochromatosis and aceruloplasminemia. Hematology service was consulted; however, workups, including LDH (1,000 U/L), haptoglobin (34 mg/dL), and serum copper (93 μg/dL), were generally inconclusive as summarized in Table 1.

**Table 1:** Laboratory findings on admission and during anemia workup.

Laboratory test	Patient value	Reference range
Serum alcohol	53.5 mg/dL	<=10 mg/dL
Serum ammonia	62 µmol/L	11-32 µmol/L
Aspartate aminotransferase (AST)	542 U/L	13-41 U/L
Alanine aminotransferase (ALT)	185 U/L	10-43 U/L
Alkaline phosphatase (ALP)	428 U/L	42-119 U/L
Total bilirubin	22.2 mg/dL	0.2-1.2 mg/dL
Direct bilirubin	15.2 mg/dL	0.1-0.3 mg/dL
Albumin	3.1 g/dL	3.5-5.0 g/dL
International normalized ratio (INR)	1.85	0.9-1.1
Hemoglobin	11.4 g/dL	12.0-16.0 g/dL (female)
Mean corpuscular volume (MCV)	97.9 fL	81-98 fL
Platelet count	175 ×10³/µL	140-450 ×10³/µL
Ferritin	17,741 ng/mL	10-291 ng/mL
Serum iron	178 µg/dL	50-170 μg/dL
Lactate dehydrogenase	1,000 U/L	140-280 U/L
Haptoglobin	34 mg/dL	40-200 mg/dL
Serum copper	93 μg/dL	70-140 μg/dL

**Figure 1:**
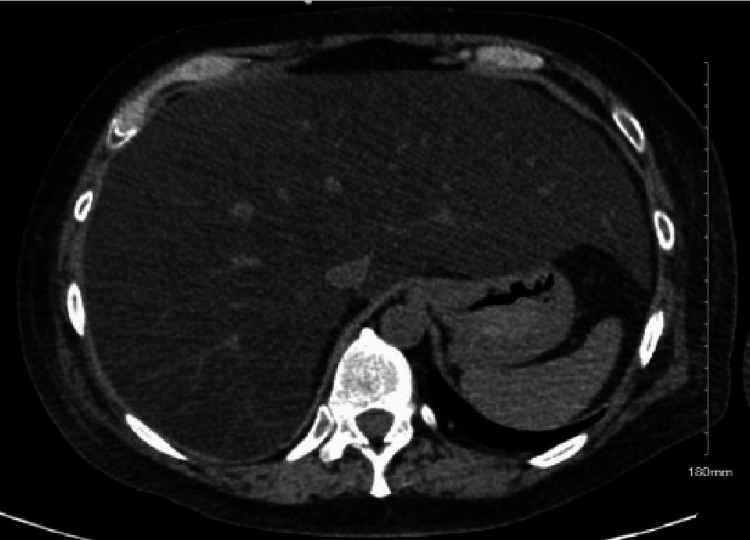
CT abdomen revealing hepatomegaly.

Abdominal ultrasound and CT scan of the abdomen were done, which showed marked hepatomegaly of 27 cm, no discrete hypodensity, with no evidence of intra- or extrahepatic ductal dilatation nor other significant findings (Figure 1).

Eventually, the patient was found to be positive for a heterozygous H63D variant on the HFE gene analysis, and a diagnosis of hereditary hemochromatosis was ascertained. Her liver function tests improved gradually throughout her admission, and her symptoms have also improved; she was discharged home with instructions for close follow-up. 

**Figure 2:**
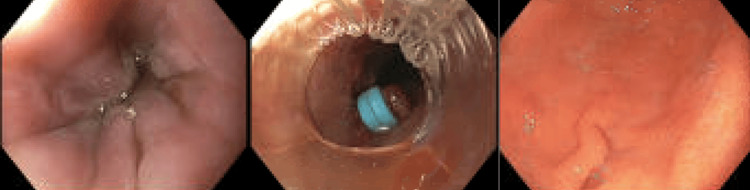
Grade II varices in the lower third of the esophagus (left), an ulcer in the middle third of the esophagus status post banding (center), and portal hypertensive gastropathy in the gastric body (right).

However, five days post-discharge, she returned with shortness of breath and left lower extremity swelling and tenderness. Chest X-ray was unremarkable. Duplex ultrasound showed no deep vein thrombosis but revealed complex collections in the left thigh suggestive of hematomas. She also reported hematochezia since discharge. During hospitalization, hemoglobin levels dropped to as low as 6.1 g/dL, requiring multiple blood transfusions. Gastrointestinal (GI) evaluation with EGD revealed a mid-esophageal ulcer with adherent clot, grade II distal esophageal varices, and diffuse portal hypertensive gastropathy (Figure 2).

**Figure 3:**
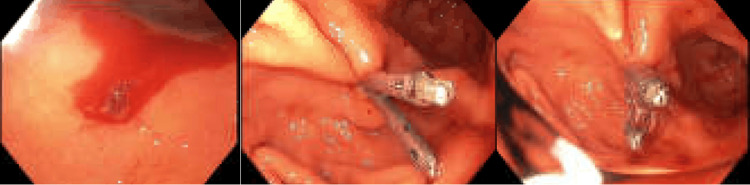
Dieulafoy lesion.

Ongoing melena with a persistent drop in hemoglobin prompted repeat EGD, revealing an actively bleeding Dieulafoy lesion in the gastric body (Figure 3), which was treated endoscopically. 

**Figure 4:**
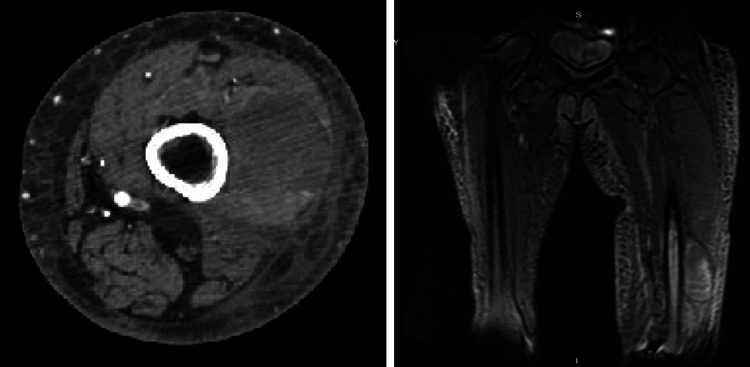
Computed tomography angiography (CTA) of the left lower extremity (left) and magnetic resonance imaging (MRI) of the femur (right) demonstrating a left vastus lateralis hematoma.

An octreotide drip was initiated for five days. Despite intervention, hemoglobin continued to fall, and thigh swelling persisted despite conservative measures. Computed tomography angiography (CTA) of the left lower extremity revealed a large intramuscular hematoma in the left vastus lateralis (Figure 4).

Interventional radiology placed a drain for hematoma evacuation, which was removed after nine days, with her hemoglobin and blood pressure remaining stable. She was ultimately discharged to a subacute rehabilitation facility for alcohol detoxification with plans for close follow-up with her primary care physician, gastroenterology, and hepatology.

## Discussion

This case highlights the interplay between severe alcoholic hepatitis and heterozygous H63D HFE mutation, presenting with extreme hyperferritinemia, multiple GI bleeding events, and spontaneous intramuscular hematoma formation.

Ferritin, an acute-phase reactant, can be markedly elevated in acute inflammatory states such as alcoholic hepatitis. Levels >10,000 ng/mL are unusual and may raise suspicion of alternative or concomitant diagnoses, such as hemophagocytic lymphohistiocytosis, Still’s disease, or iron overload syndromes [5]. In this patient, the extreme ferritin elevation (17,741 ng/mL) may have reflected a combination of acute hepatic inflammation, alcohol-induced liver injury, and impaired iron metabolism due to the H63D HFE mutation. Although heterozygous H63D carriers generally do not develop clinically significant iron overload compared with C282Y homozygotes or C282Y/H63D compound heterozygotes [1,2], alcohol use can act synergistically to exacerbate iron accumulation and hepatic injury [6].

The patient’s GI bleeding was multifactorial. Endoscopic findings of an esophageal ulcer, esophageal varices, portal hypertensive gastropathy, and a gastric Dieulafoy lesion underscore the broad spectrum of hemorrhagic complications that can occur in advanced liver disease. Dieulafoy lesions, in particular, are rare vascular anomalies that account for 1%-2% of cases of acute GI bleeding [7]. Their presence in this patient further complicated management, requiring repeat endoscopy and endoscopic therapy.

In addition, the spontaneous intramuscular hematoma in the vastus lateralis represents an unusual but clinically significant complication in cirrhotic patients. Hemostatic imbalance in advanced liver disease results from a combination of thrombocytopenia, impaired synthesis of clotting factors, and increased fibrinolysis [8]. Although spontaneous muscle hematomas are more frequently reported in patients receiving anticoagulation, they can also arise in the setting of coagulopathy and portal hypertension. In this case, hematoma formation further contributed to the patient’s transfusion requirements and prolonged hospitalization, necessitating interventional radiology drainage.

Management of this patient was complex, requiring a multidisciplinary approach involving hepatology, gastroenterology, and interventional radiology. Initial treatment of severe alcoholic hepatitis with corticosteroids was discontinued early due to poor response on the Lille scoring, consistent with guidelines [3]. Supportive measures, alcohol withdrawal management, nutritional support, and close follow-up remain essential pillars of care. Notably, the identification of the H63D mutation emphasizes the need for continued monitoring of iron indices, though the clinical significance of heterozygosity in isolation remains debated.

This case illustrates the compounded hepatic and systemic effects of heavy alcohol use and genetic susceptibility to altered iron metabolism. It also highlights rare but essential complications, including Dieulafoy lesion bleeding and spontaneous intramuscular hematoma, which can significantly increase morbidity in patients with severe alcoholic hepatitis.

## Conclusions

Elevated ferritin levels are a non-specific finding that is often seen in acute or chronic inflammation, alcoholic liver disease, or iron overload disorders. This case underscores the complex interaction between alcohol-induced liver injury and genetic susceptibility from a heterozygous H63D mutation, leading to extreme hyperferritinemia and severe clinical decompensation. It highlights the importance of considering both acute inflammatory states and underlying genetic factors when interpreting markedly elevated ferritin levels. Additionally, it demonstrates the broad spectrum of hemorrhagic complications in advanced liver disease, including rare entities such as Dieulafoy lesion bleeding and spontaneous intramuscular hematoma. Early recognition, multidisciplinary management, and close follow-up are crucial to improving outcomes in these high-risk patients.
